# Clinical Comparison of Autogenous Bone Graft with and without Plasma Rich in Growth Factors in the Treatment of Grade II Furcation Involvement of Mandibular Molars

**DOI:** 10.5681/joddd.2013.004

**Published:** 2013-02-21

**Authors:** Ardeshir Lafzi, Adileh Shirmohammadi, Masoumeh Faramarzi, Sahar Jabali, Arman Shayan

**Affiliations:** ^1^Professor, Department of Peiodontics, Faculty of Dentistry, Shahid Beheshti University of Medical Sciences, Tehran, Iran; ^2^Dental and Periodontal Research Center, Tabriz University of Medical Sciences, Tabriz, Iran; ^3^Associate Professor, Department of Periodontics, Faculty of Dentistry,Tabriz University of Medical Sciences, Tabriz, Iran; ^4^Assistant Professor, Department of Periodontics, Faculty of Dentistry, Tabriz University of Medical Sciences, Tabriz, Iran; ^5^Postgraduate Student, Department of Periodontics, Faculty of Dentistry, Tabriz University of Medical Sciences, Tabriz, Iran; ^6^Postgraduate Student, Department of Orthdontics, Faculty of Dentistry, Shahid Beheshti University of Medical Sciences, Tehran, Iran

**Keywords:** Bone grafting, furcation defect, periodontal regeneration, platelet-derived growth factor

## Abstract

**Background and aims:**

Plasma rich in growth factors (PRGF) is a concentrated suspension of growth factors, which is used to promote periodontal tissue regeneration. The aim of this randomized, controlled, clinical trial was to evaluate of the treatment of grade II mandibular molar furcation involvement using autogenous bone graft with and without PRGF.

**Materials and methods:**

In this double-blind clinical trial, thirty mandibular molars with grade II furcation involvement in 30 patients were selected. The test group received bone graft combined with PRGF, while the control group was treated with bone graft only. Clinical parameters included clinical probing depth (CPD), vertical clinical attachment level (V-CAL), horizontal clinical attachment level (H-CAL), location of gingival margin (LGM), surgically exposed horizontal probing depth of bony defect (E-HPD), vertical depth of bone crest (V-DBC), vertical depth of the base of bony defect (V-DBD), and length of the intrabony defect (LID). After six months, a re-entry surgery was performed. Data were analyzed by SPSS 14, using Kolmogorov, Mann-Whitney U, and paired t-test.

**Results:**

After 6 months, both treatment methods led to significant improvement in V-CAL and H-CAL and significant decreases in CPD, E-HPD, V-DBD and LID; there was no significant difference in LGM and V-DBC in any of the treated groups compared to the baseline values. Also, none of the parameters showed significant differences between the study groups.

**Conclusion:**

Although autogenous bone grafts, with or without PRGF, were successful in treating grade II furcation involvement, no differences between the study groups were observed.

## Introduction

Inflammation of tooth-supporting tissues, including cementum, periodontal fibers and alveolar bone, causes periodontal disease. The ultimate goal of periodontal treatment is the regeneration of periodontal tissues lost during the disease process. A variety of treatment modalities, including the use of autogenous bone grafts (ABG) and bone substitute materials,^1,2^ guided tissue regeneration (GTR) with the use of barrier membranes,^3^ and growth factors,^4^ have been proposed to promote regeneration of periodontal tissues. It would be more difficult to treat if the inflammation progresses into the furcation of multi-rooted teeth.^5^ Treatment of furcation involvement is still a challenge in periodontal treatment because of the specific anatomical features and different structural details in each tooth, and to date, no definitive solution has been found.^6^

GTR procedures help regenerate intrabony defects and peri-implant defects, grade II furcation involvement in mandibular teeth, and increase bone height in edentulous areas. Although it is useful in the treatment of grade II maxillary furcation involvement and grade III furcation defects of animals,^4,7,8^ the results of human studies are not predictable.^9^

Recently, a new path has been found in periodontal therapy based on the utilizing of growth factors and the principles of tissue engineering. These factors combined with the common method have provided new procedure called growth factor modulated GTR.^10^

Cho et al^10^ and Park et al^11^ indicated that grade III furcation defects in beagle dogs are filled with new bone up to 80% and new periodontal ligament (PDL) up to 20% after PDGF (platelet-derived growth factor)-modulated GTR therapy, whereas these amounts after GTR therapy alone are 14.6% and 4.6%, respectively. Introduction of the platelet-rich plasma (PRP) as a source of growth factors such as PDGF, TGF-ß and IGF-I prompted its use as an autogenic and safe material in the enhancement of regeneration process.^12,13^

The healing of wounds caused by periodontal surgery is complicated, and different cells and growth factors are involved in the healing process.^14^ Studies on wound healing have shown that, after surgery, platelets participate in clot forming and release a series of growth factors involved in cell division and differentiation, consequently in the repair and tissue forming.^15^ Therefore, these growth factors can be used to enhance tissue healing process. Recently, plasma rich in growth factors (PRGF) which is a concentrated suspension of growth factors has been used to promote healing and periodontal tissue regeneration.^15,16^ PRGF (plasma rich growth factors) is a source of autologous growth factors, consisting of PDGF and transforming growth factor-ß (TGF-ß).^16^ Mansouri et al^17^ found that application of a combined technique using BPBM (bovine porous bone mineral)/PRGF, compared to the BPBM alone, results in greater healing, although not significant, in the treatment of mandibular class II furcation defects. The present study compared the clinical outcomes obtained by the combination of ABG and PRGF with those obtained by ABG in the regeneration of grade II furcation involvement of human mandibular molars.

## Materials and Methods

### Study Population and Experimental Design

In this double-blind clinical trial, 30 mandibular molars with horizontal probing depth of ≥ 3 mm (grade II furcation involvement based on Hamp classification) from 30 patients, who had referred to the Department of Periodontics, Tabriz University of Medical Sciences, for the treatment of periodontal disease, were included. The nature of this investigation was explained in detail and the patients signed an informed consent form for the initial surgery and also the re-entry to evaluate the treatment results and correct the probable remaining defects.

### Exclusion Criteria

- Pregnancy or lactation for women
- Serious kidney/liver problems or diabetes
- Smoking, use of alcohol or narcotic drugs
- Systemic diseases affecting the periodontium and the use of drugs effective on periodontal healing such as corticosteroids
- Use of antibiotics during the previous two months and no long-term treatment with NSAIDs
- Need for prophylaxis prior to surgery

### Inclusion Criteria

- Presence of moderate to severe chronic periodontal disease by clinical and radiographic evidence; presence of buccal grade II furcation involvement of mandibular molars by radiographic and clinical evidence; Furcation defect at least 3 mm in depth (and thus, in general, surpassing half of the buccolingual thickness of the tooth) but not through-and-through, i.e. there is still some interradicular bone attached to the angle of the furcation (cul-de-sac furcation defect).
- Tooth vitality and no periodontal or endodontic lesions in the selected tooth.
- The proof of blood safety by Hbs Ag, HCV, and HIV tests.

### Preoperative Measurements

All the patients underwent initial therapy and were monitored until the plaque index (O'leary Plaque Index) was less than 20% and remained stable.

At the time of surgery, clinical parameters were measured with a Williams periodontal probe (PWD, Hu-Friedy Immunity, USA) from a fixed point using customized acrylic stents to prevent angulation and positioning errors. The degree of tooth mobility was determined and tooth radiograph was obtained.

### PRGF Preparation

In the test group, on the surgery day 10 mL of blood was taken before surgery and was transferred to 5-mL glass tubes containing 3.8% trisodium citrate, which is an anticoagulant. Then, these glass tubes were centrifuged at 280 G speed for 7 minutes at room temperature so that blood was divided into four components:

- Plasma without growth factors located on the uppermost layer, which was about 0.5 ml in volume;
- Plasma with growth factors with a volume of about 0.5 ml;
- PRGF with a volume of approximately 0.5 ml;
- Red blood cells;

PRGF is the utilizable component, which is located above the red blood cells.^18^

### Measurements

All baseline clinical parameters were obtained on the day of the surgery by one examiner, who was blind to the type of treatment. Final parameters were taken 6 months postoperatively by the same examiner, again blind to the method of the study. A calibration exercise was performed to obtain acceptable intra-examiner reproducibility for probing depth and recession of the gingival margin. Prior to the study and after 6 months, five patients each with ten teeth, with a probing depth of > 4 mm on at least one aspect of each tooth were used for calibration. The examiner evaluated the patients on two occasions, 48 hours apart. Calibration was accepted if > 90% of the recording could be reproduced within a 1.0-mm difference.

The following soft tissue measurements before surgery and six months after the treatment were included:

- Clinical probing depth (CPD): Margin of gingiva to the base of periodontal pocket.
- Vertical clinical attachment level (VCAL): Inferior margin of the stent to the base of periodontal pocket depth.
- Horizontal clinical attachment level (H-CAL): Inferior margin of the stent to the deepest probing area of furcation in the horizontal direction.
- Location of gingival margins (LGM): Inferior margin of the stent to the gingival margin.

### Surgical Procedure

**Figure 1:**
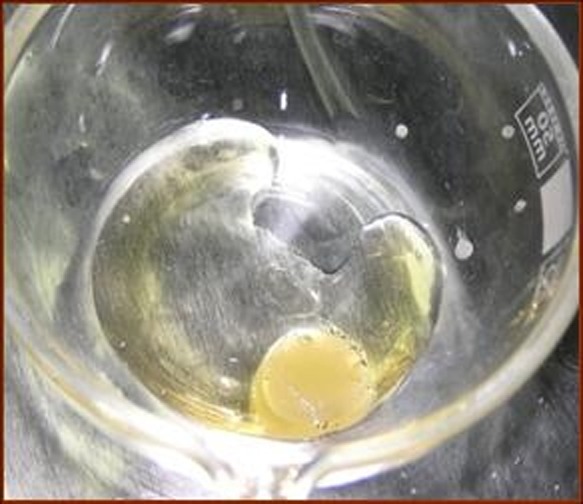
Prepared PRGF.

Each case of furcation defect, based on the table of randomized data, was treated with either ABG or ABG accompanied by PRGF (ABG/PRGF) methods; therefore, in each of the ABG and ABG/PRGF groups, 15 patients were enrolled. In the ABG group, surgical sites were anesthetized utilizing 2% lidocaine with 1:80,000 epinephrine. Following buccal and lingual sulcular incisions, full-thickness flaps were elevated. Using a surgical blade, the muscle insertions from the flap were dissected in order to enhance the coronal displacement of the flap to achieve a tension-free full-coverage of defects. Granulation tissue was removed to allow visualization of the defect. Root surfaces and furcation fornix were scaled and root planed by means of hand instruments or ultrasonic devices. No osteotomy of the tooth-supporting bone was carried out, but osteoplasty was performed when indicated. Autogenous bone chips were retrieved from adjacent areas and as far as possible, they were made smaller in size by means of hand instruments. The defects were overfilled with bone grafts and then tightly packed using amalgam condensers to fill through of furcation defect. Closure was accomplished using 4-0 silk sutures and a periodontal dressing was used. In the ABG/PRGF group, all stages including the use of ABG/PRGF were similar except for PRGF preparation procedure and mixing with autogenous bone. PRGF (Figure 1) was prepared by adding one ml of the provided PRGF to 50 microliters of 10% calcium chloride (citrate inhibitor that causes coagulation of plasma) and a semi-solid mass was obtained which was easy to use in the surgical site. Clotting time at room temperature is 5-8 minutes.^18^ After preparation of the autogenous bone—the same way as mentioned for ABG groups—the bone chips were immersed in the prepared PRGF to infuse growth factors into the bone.^19^

Hard tissue measurements were made with the same stent:

- Horizontal probing depth of the bony defect surgically exposed (E-HPD): The horizontal distance between the deepest probing sites of furcation to the installed rubber stop on probe which was in contact with the root prominence while the probe was perpendicular to the tooth long axis.
- Vertical depth of the bone crest (V-DBC): Inferior margin of the stent to the bone crest.
- Vertical depth of the base of bony defect (V-DBD): Inferior margin of the stent to base of the bony defect.
- Length of the intrabony defect (LID): Alveolar crest to the base of bony defect.

### Postoperative Care

**Figure 2:**
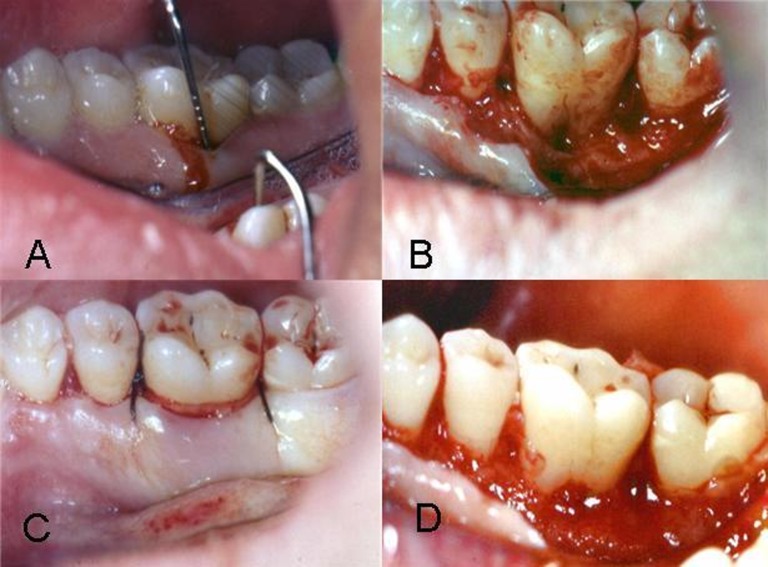
Furcation treatment in ABG/PRGF group: A. Before surgery; B. Furcation defect after flap preparation; C. Filling of defect with the bone chips and PRGF; D. Six months after surgery.

All subjects received postoperative instructions, including rinsing with 0.2% chlorhexidine (twice daily for 2 weeks), as well as amoxicillin and ibuprofen medications for 1 week. The patients were advised to avoid brushing the area up to six weeks after surgery. The patients were re-visited after 10 days for removal of the periodontal dressing and sutures. Patients were controlled weekly during the first month and then monthly until the sixth month and received professional prophylaxis and oral hygiene reinforcement. Six months after the primary surgery, a re-entry surgery was performed. The re-entry procedure was aimed at correction of any remaining defects and evaluation of the results of the treatment with reference to the soft and hard tissue parameters. The method of re-entry measurements was similar to primary measurements. Figure 2 shows treatment of furcation in one ABG/PRGF group.

### Statistical Analysis

The patient was defined as the statistical unit. The normality of data distribution was evaluated using Kolmogorov–Smirnov test. Analysis was performed using descriptive statistical methods (mean ± SD) and paired t-test. Mann-Whitney U test was used for comparison of intra-group differences at baseline and 6 months postoperatively. SPSS 14 software was employed. In the present study, statistical significance was defined at P < 0.05.

## Results

Only two defects were lingual furcation defects. Postoperative healing was uneventful in all cases. All of the subjects returned for re-evaluation after 6 months, and maintained excellent levels of oral hygiene. Plaque and gingival indexes did not demonstrate any significant differences compared to baseline values.

There were no significant differences in the initial measurements of soft and hard tissue parameters between the ABG and ABG/PRGF groups (Tables 1 and 2), so the differences observed at the end of study were attributed to the direct effect of the treatment protocol.

**Table 1:** Soft tissue parameters at baseline and after 6 months

Variable	Group	Baseline value (mm)	Baseline comparison	6-monthvalue (mm)	Intra-group comparison	Inter-group comparison
CPD	ABG	4.86±0.74		2.40±0.50	P<0.001*	
	ABG/PRGF	4.66±0.72	P=0.48	2.33±0.48	P<0.001*	P=0.77
V-CAL	ABG	11.13±1.18		8.46±1.24	P<0.001*	
	ABG/PRGF	10.8±1.08	P=0.48	8.26±1.16	P<0.001*	P=0.56
H-CAL	ABG	4±0.65		2.60±0.63	P<0.001*	
	ABG/PRGF	4.6±0.59	P=0.80	2.46±0.51	P<0.001*	P=0.65
LCM	ABG	6.26±0.96		6.06±0.96	P=0.42	
	ABG/PRGF	6.31±0.83	P=0.77	5.93±0.88	P=0.38	P=0.59

Asterisk indicates statistical significance based on P < 0.05.

**Table 2:** Hard tissue parameters at baseline and after 6 months

Variable	Group	Baseline value (mm)	Baseline comparison	6-month value (mm)	Intra-group comparison	Inter-group comparison
	ABG	4.13±0.51		3.26±0.45	P<0.001*	
E-HPD	ABG/PRGF	4±0.37	P=0.56	3±0.37	P<0.001*	P=0.25
	ABG	6±1.06		6.13±1.88	P=1.000*	
V-DBC	ABG/PRGF	5.8±0.94	P=0.41	5.53±1.72	P=0.21	P=0.41
	ABG	10.06±1.43		8.46±1.50	P<0.001*	
V-DBD	ABG/PRGF	9.66±1.72	P=0.48	8.13±1.24	P<0.001*	P=0.68
	ABG	3.93±0.59		2.33±1.29	P<0.001*	
LID	ABG/PRGF	3.86±O.51	P=0.80	2.60±1.40	P=0.002*	P=0.68

Asterisk indicates statistical significance based on P < 0.05.

### Soft Tissue Parameters

Soft tissue parameters are presented in Table 1. In comparison with the baseline data, both the test and control groups showed statistically significant differences (P < 0.001). Clinical probing depth reductions in the ABG group were 2.46 mm and 2.33 mm in the ABG/PRGF group. However, the inter-group differences after 6 months were not statistically significant (P = 0.77). The reductions of vertical clinical attachment levels were 2.67 mm and 2.54 mm in the ABG and ABG/PRGF groups, respectively. Both intra-group differences were significant (P < 0.001); however, there were no statistically significant differences between the groups 6 months postoperatively (P = 0.56). The reductions of horizontal clinical attachment levels were 1.40 mm and 2.14 mm in the ABG and ABG/PRGF groups, respectively. Both intra-group differences were significant (P <0.001), but the inter-group differences after 6 months were not statistically significant (P = 0.65). Average changes of 0.38 mm and 0.2 mm were observed in the location of gingival margin between the baseline and 6-month data in the test (P = 0.38) and control (P = 0.42) groups. The differences in each group and between the groups after 6 months were not statistically significant (P > 0.05).

### Hard Tissue Parameters

Hard tissue parameters are presented in Table 2. Evaluation of the hard tissue findings indicated that reductions of surgically exposed horizontal probing depths of bony defects in the ABG and ABG/PRGF groups were 0.87 mm and 1 mm, respectively. In comparison with the baseline, the differences in both groups were statistically significant (P < 0.001); however, there were no statistically significant differences between the groups (P = 0.25).

Vertical depths of alveolar crests in the ABG and ABG/PRGF groups showed 0.13 mm and 0.32 mm reductions, respectively. In comparison with the baseline data, the differences in both groups and also between the groups were not statistically significant (P > 0.05).

An evaluation of the hard tissue findings indicated that both treatment modalities resulted in defect fills when baseline data and 6-month data were compared. The ABG group showed 1.6 mm (P < 0.001) of filling of bony defects (V-DBD), while this was 1.53 mm in the ABG/PRGF group (P < 0.001); however, after 6 months, the inter-group differences were not statistically significant (P = 0.68).

After 6 months, the length of intrabony defect in the ABG and ABG/PRGF groups diminished 1.60 mm and 1.26 mm, respectively. Both the intra-group differences were significant (P < 0.001); however the inter-group differences after 6 months were not statistically significant (P = 0.68).

## Discussion

T Bottom of FormtTTThe present study was undertaken to evaluate the clinical effectiveness of autogenous bone graft alone or combined with PRGF in the regeneration of grade II furcation involvement of mandibular molars. Autogenous bone itself has an osteoinductive and osteoconductive role and therefore it is considered a gold standard.^20^ Thirty mandibular molars with grade II furcation involvement were treated. The control group was treated with ABG, while in the test group ABG/PRGF was used. Postoperative healing was uneventful in all the cases, and no complication was observed. This confirms that both treatment modalities are tolerated by periodontal tissues without any side effects on the healing process. Plaque and gingival index values ​​at the start of the study indicated good plaque control of the patients and absence of at least gingival inflammation. These conditions continued until the end of the study.

At baseline, soft and hard tissue clinical parameters did not show significant differences in both groups; therefore, it can be interfered that the observed differences resulted from the direct effect of the treatment modality. The reduction of clinical probing depth (CPD) is one of the important goals of periodontal therapy. Treatment with ABG/PRGF and ABG caused 2.33 mm and 2.64 mm reductions in probing depths, respectively; i.e. both treatments could effectively reduce probing depths.

Vertical clinical attachment losses (V-CAL) in the ABG/PRGF and ABG groups were 2.54 and 2.67 mm, respectively, which showed significant in-group differences, but no significant between group difference. This finding suggests that the gain in attachment is the major contributor to the reduction in the probing depth in both groups, and changes in the position of gingival margin involved a small part of the reduction. Absence of significant differences in the location of gingival margin (LGM) after surgery in both groups confirms this notion. These amounts ​​in the test and control groups were 0.19 mm and 0.2 mm, respectively. Vertical attachment gain in both groups of this study was greater than the study results of Donos et al,^21^ who reported it to be approximately 1.35 mm, but it was similar to the results of a study by Donos et al,^19^ with 2.3 mm. Attachment gain might result from true periodontal regeneration or defect healing by new connective tissue attachment or long junctional epithelium. To determine the nature of the gained attachment, histological studies are necessary. According to a histological study by Yukna et al,^22^ after six months out of the 10 defects treated with EMD, in 3 cases a real regeneration, in 3 cases connective tissue attachment and in 4 cases the long junctional epithelium were observed.

H-CAL in the ABG/PRGF and ABG groups were 1.5 and 1.4 mm, respectively. However, it was significant in both groups, but there were no significant differences between the groups. Horizontal attachment gain in both groups of the present study was similar to that in a study by Donos et al,^21^ in which it was estimated to be 1.4 mm in the buccal furcation and 0.5 mm in the lingual furcation. Improved H-CAL demonstrates that the treated defect is resistant to probe penetration. Re-entry surgery can be used for better evaluation of bone regeneration and the defect repair by direct observation of the site. One of the desirable results of regenerative treatment is bone filling of the defect. Since vertical and horizontal attachment gain necessarily does not mean bone formation, re-entry surgery was performed to investigate whether bone formation had been established.

In re-entry, the fillings of intrabony defect (V-DBD) in the ABG/PRGF and ABG groups were 1.5 mm and 1.6 mm, respectively. Both the intra-group differences were significant; however there were no significant differences between the groups, suggesting that both treatment protocols had significantly resulted in a greater amount of intrabony defect fill.

Vertical depths of bone crest in the test and control groups decreased 0.32 mm and 0.13 mm, respectively. Compared to the baseline data, both intra-group differences were not significant. Also, the differences between the groups were not statistically significant. The amount of crestal bone changes in the test group of the present study were in contrast to Forum et al^23^ findings in relation to defects treated with EMD; crestal bone loss was 0.46 mm.

Changes in the length of intrabony defect (LID) are affected by the amount of defect fill and the crestal bone change. In this study the intrabony defect depth as observed 6 months postoperatively decreased 1.26 mm and 1.6 mm in the test and control groups, respectively. These differences were significant in both groups, but they were not significant between the groups. Since crestal bone loss was negligible, these changes are mainly due to defect fill.

E-HPD in the ABG/PRGF and ABG groups decreased 1 mm and 0.87 mm, respectively. Compared to the baseline data, these differences were significant in both groups, but they were not significant between the groups. It indicates that the two treatment modalities led to significant horizontal bone fill. Jepsen et al^24^ studied E-HPD in grade II furcation involvement following treatment with EMD. They reported that E-HPD decreased 2.6 mm during re-entry. This amount is greater than the measured value in the present study. The observed new tissue in the furcation area at the time of re-entry was a rubbery tissue and did not permit the entrance of a probe.

Full closure of furcation, which is considered an ideal result of regenerative treatments, is the most important criteria of treatment success aiming to improve the prognosis of desired tooth.

Unfortunately, due to the varying anatomy of furcation area in different teeth, it is not possible to consider it an objective method to determine furcation closure.

## Conclusion

Both treatment modalities in the present study resulted in clinically significant improvements in all the soft and hard tissue parameters when baseline data and 6-month data were compared. Nonetheless, there were no significant differences between these methods in the treatment of grade II furcation involvement of mandibular molars.
